# What Do the Hospital Pharmacists Think about the Quality of Pharmaceutical Care Services in a Pakistani Province? A Mixed Methodology Study

**DOI:** 10.1155/2015/756180

**Published:** 2015-01-08

**Authors:** Ghulam Murtaza, Rozina Kousar, Saira Azhar, Shujaat Ali Khan, Qaisar Mahmood

**Affiliations:** ^1^Department of Pharmacy, COMSATS Institute of Information Technology, Abbottabad 22060, Pakistan; ^2^Department of Environmental Sciences, COMSATS Institute of Information Technology, Abbottabad 22060, Pakistan

## Abstract

The objective of this study was to evaluate the perception of hospital pharmacists regarding quality of pharmaceutical care services in Khyber Pakhtunkhwa (KPK) Province, Pakistan, through qualitative as well as quantitative approach. For qualitative study, snow ball sampling technique was used. In quantitative part, a cross-sectional study was conducted in 112 hospital pharmacists (out of 128 accessed ones) from both private and public hospitals in six major divisions (divisions are the third tier of government in Pakistan, between the provinces and districts) of KPK. The qualitative study yielded five major themes during thematic analysis: (a) patients reporting, (b) lack of patient counseling, (c) lack of participation in health awareness programs, (d) pharmacists reducing the prescribing errors, and (e) insufficient number of pharmacists. A great proportion (67.9%) of the pharmacists was unsatisfied with their participation in health awareness programs. Findings of both phases revealed that hospital pharmacists in Pakistan are not actively participating in the provision of pharmaceutical care services. They are facing various hurdles for their active participation in patient care; major obstacles include the unavailability of sufficient number of pharmacists, lack of appropriate time for patient counseling, and poor relationship between pharmacists and other health care providers.

## 1. Introduction

The pharmacy profession is undergoing a paradigm shift from product-oriented to patient-oriented practice. This patient-oriented practice is termed as pharmaceutical care. Throughout the world, the principal task of pharmacy is considered to be the pharmaceutical care [1]. Pharmaceutical care means the responsible provision of drug therapy for achieving definite outcomes that improve the quality of patient's life [2]. Improved therapeutic outcomes include greater patient safety, better drug therapy and disease management, valuable health care expenditure, best adherence, and improved quality of life [3].

Pharmaceutical care practice involves a covenantal relationship between the pharmacist and the patient in which drug use is controlled by the pharmacist along with commitment and understanding of the patient's interest [2]. It is perceived as pharmacy profession's growth by accepting the social duty to diminish preventable drug-related morbidity and mortality [4]. Pharmaceutical care practice involves the periodic revolution in health care services provided by pharmacists. Several studies conducted in the developed countries have reported that pharmaceutical care practice has a considerable positive effect on health care cost and management [1] and pharmacists have worked a lot for implementation of pharmaceutical care practice. Additionally, it is considered that pharmacists can play a great role in the fundamental health care provision, particularly in economically developing countries. But in developing countries, implementation of pharmaceutical care still encounters a number of hurdles, among which are time constraints, absence of recognized reimbursement system, unavailability of appropriate space, less access to patient medication record, insufficient number of competent pharmacists, and shortage of standard practice strategy [5, 6]. As an innovative practice, most of the professionals feel reluctant to its implementation. Some pharmacists disagree to the observation that the implementation of pharmaceutical care services has exercised potential positive effects on the therapeutic outcomes [7], while other pharmacists are of the opinion that pharmacy future relies upon the provision of other healthcare services also rather than just dispensing [8]. For successful execution of pharmaceutical care, the pharmacist's philosophy of practice holds vital importance. Moreover, pharmacists need to adapt their knowledge, attitude, and skills to this new practice, which integrates traditional knowledge of pharmaceutical science with clinical aspects of patient care, namely management skills, ability to actively communicate with medical teams, and competence to resolve drug-related problems [9].

For improvement of medicines' use and patients' quality of life, health authorities in Pakistan are needed to execute pharmaceutical care practice in the healthcare system of the country. There is a shortage of pharmacists in private as well as public healthcare sectors but for the past few years their number has been increasing to an acceptable extent. In spite of their involvement in direct patient care, hospital pharmacists are more involved in administrative activities [10]. The objective of this study was to assess the hospital pharmacists views regarding the quality of pharmaceutical care services provided to patients in hospitals of Khyber Pakhtunkhwa (KPK) Province, Pakistan.

## 2. Methods

This study was conducted both qualitatively and quantitatively after getting approval from Departmental Research Ethical Committee. In qualitative phase, an interview guide was developed after extensively reviewing the literature [1, 5, 9, 11] for execution of interviews. After getting appointment telephonically, the participants were interviewed directly by one of the researchers (RK), who is pharmacist, by using snow ball sampling technique in hospital pharmacies. Saturation point was reached after 13th interview.

In quantitative phase of this study, questionnaire was developed for this cross-sectional survey on the basis of findings of qualitative phase. Moreover, literature was extensively reviewed [5, 9, 12, 13]. To assess the face validity of the questionnaire, the participants were selected. These participants were asked for their views on the significance, worth, and simplicity of each question and to identify which question(s) they would point out “to be removed” in order to make the questionnaire brief. In addition, the participants were also welcomed to suggest further comments on the questions whether they were understandable or not. Most of them suggested simplifying the questions. The reliability test was applied to all the variables comprising all domains. The reliability of the tool was estimated on the basis of Cronbach's Alpha ($\overset{´}{\alpha }=0.60$). After development of questionnaire, self-administered survey was conducted by data collection team in six major divisions of KPK Province, in which eight most populous cities were targeted including Peshawar, Mardan, Dera Ismail Khan, Bannu, Malakand, Haripur, Abbottabad, and Mansehra. The questionnaire had six sections: (i) demographic profile, (ii) pharmacist's interaction with doctors, (iii) perception regarding patient counseling, (iv) patient reporting of ADRs, (v) participation in health awareness programs, and (vi) perception regarding reducing prescribing errors.

For collection of data, nonprobability convenient sampling was adopted due to the unavailability of a database showing the exact number of hospital pharmacists. To overcome this, before the data collection, a survey was carried out to locate the hospitals with hospital pharmacists. A total of 128 hospital pharmacists were identified in 18 hospitals of these eight cities.

### 2.1. Statistical Analysis

The data was analyzed by using Statistical Package for Social Sciences (SPSS version 17). The results of each question were reported as frequencies and percentages. For testing significant association among the dependent variables (perception regarding patient counseling, reducing prescribing errors, patients reporting of ADRs, and participation in health awareness programs) and independent variables (age range, gender, years of practice, and type of hospital), Chi-square test was used. Statistical significance was accepted at *P* value of ≤0.05.

## 3. Results and Discussion

In qualitative and quantitative phases, age of the interviewees ranged between 20 and 50 years.

Thematic content analysis of collected data yielded 5 major themes: (a) patients reporting, (b) lack of patient counseling, (c) lack of participation in health awareness programs, (d) pharmacists reducing the prescribing errors, and (e) insufficient number of pharmacists.

### 3.1. Theme 1: Patient's Reporting

Almost all the respondents are of the view that the patients are not informing hospital pharmacists about adverse reaction occurrence.

*“Patients are unaware of their rights; they are not informing us about the adverse drug effects”** (HP2)**.*

*“Patients never inform us about ADR. They are trusting on [sic] their doctors, so they contact to [sic] them for such matters”** (HP6)**.*

*“I will say rarely, just those patients who know about the role of a pharmacist. Only they came to us for reporting of adverse drug effects”** (HP9)**. *

### 3.2. Theme 2: Lack of Patient Counseling

Although the pharmacist is the person who can counsel the patients more appropriately, a large number of pharmacists are limited to drug procurement.

*“We are not involved in direct patient care. If the patients come to us, we guide them. Otherwise, we are not involved in patient counseling due to lack of time and enough pharmacy staff”** (HP5)**.*

*“We are counseling the patients on their demand, when they ask about drug-related matters; we provide them information and guide them properly”** (HP12)**.*

*“I do my best to counsel each patient properly, but it is a difficult task due to the work load and lack of sufficient time”** (HP11)**. *

*“We are not involved in patient counseling, progress report monitoring and development of a drug therapy plan for the patient. Physicians are not ready to accept the pharmacists while patients are not aware of pharmacists”** (HP8)**. *

### 3.3. Theme 3: Lack of Participation in Health Awareness Programs

Most of the pharmacists are of the opinion that health awareness programs are organized by the hospitals but the pharmacists are not appreciated for participation.

*“We are not actively involved in the organization of health awareness programs. We are just involved to an extent to arrange the pharmaceutical sponsors for such programs”** (HP1)**.*

*“Health awareness programs are organized by the hospital, but doctors are more actively involved in the organization of these programs. Pharmacists are not encouraged to participate actively”** (HP4)**.*

*“Due to the daily workload and insufficient number of pharmacists we do not find enough time to participate actively in health awareness programs”** (HP9)**.*

### 3.4. Theme 4: Pharmacists Reducing the Prescribing Errors

Majority of pharmacists believe that they are the drug experts who can reduce the prescribing errors to the greater extent.

*“By pharmacist's involvement in ward rounds, prescribing errors will be reduced to a greater extent. If one pharmacist is hired on each 20 beds [sic], then it will make the patient's health assessment easy and effective”** (HP3)**.*

*“Pharmacist is the person who knows well about the drugs and its [sic] effects. When he is involved in ward rounds, the prescribing errors such as dose adjustment, drug-drug interactions, giving of two or more drugs with same effects (those are the main reasons of ADRs) will be reduced automatically”** (HP5)**.*

*“According to me, dose calculation and adjustment are the work of a pharmacist. Once they will be involved in these activities, the chances of errors will be reduced”** (HP8)**.*

*“An error in prescription is a common practice. Doctors are prescribing some irrelevant medicines like vitamins and nutrients instead of considering and focusing on the disease. So when the pharmacist works with them, the chances of errors will be minimized”** (HP10)**.*

### 3.5. Theme 5: Insufficient Number of Pharmacists

All the respondents agreed that hospital pharmacists working in hospitals are very less in number. There should be sufficient number of pharmacists to make the implementation of the pharmaceutical care services possible.

*“We have an insufficient number of pharmacists in the hospital due to which we are limited to the drug store and have no direct access to the patients and cannot solve the patient problems”** (HP2)**.*

*“We are involved in purchasing and drug inventory due to the lack of pharmacy staff. If a minimum of one pharmacist will be available in each ward, lots of drug-related problems can be solved out”** (HP4)**.*

*“To fulfill the requirement of patient care, there is a need to hire the sufficient number of pharmacists in each hospital since very fewer numbers of pharmacists are presently working in hospitals”** (HP8)**.*

In quantitative phase, questionnaires were administered to atotal of 128 hospital pharmacists. Out of which, 112 filled questionnaires (response rate = 87.5%) were recovered from pharmacists in eight cities, Peshawar (91), Mardan (2), Dera Ismail Khan (2), Bannu (2), Malakand (1), Haripur (5), Abbottabad (8), and Mansehra (1). The demographic data of the respondents is given in Table 1.

The respondent's responses towards questions on their interaction with doctors are given in Table 2.  Table 3 indicates the perception of pharmacists regarding patient counseling. Perception regarding patient counseling is listed in Table 4. Additionally, hospital pharmacists' responses to their participation in health awareness programs are listed in Table 5. Moreover, Table 6 describes the responses of the hospital pharmacists regarding prescribing errors.

The current study was focused on the perception of hospital pharmacists regarding the execution and quality of pharmaceutical care services provided to patients in KPK Province, Pakistan. Both phases of this study identified almost similar findings. The study identified that there was a lack of provision of pharmaceutical care services to the patients in Pakistan, since the patients did not report the occurrence of adverse drug reactions to the pharmacists that supported the study conducted in Karachi [14], according to which majority of pharmacists stated that the patients are not informing them about adverse drug reaction events. The present study also revealed that there was a lack of communication among hospital pharmacists and physicians in relation to drug information that was consistent with a previous qualitative study carried out in Punjab Province of Pakistan [15]. The researchers showed that although physicians accepted the pharmacist as an essential member of the healthcare team the pharmacists were less in number which could be due to the prescriber's high proficiency or their low belief in pharmacists' capabilities. According to a majority of respondents, the main obstacle in implementation of pharmaceutical care services (especially in terms of counseling) was the lack of time that was in line with an Argentinean study [16], which also reported the lack of time as one of the barriers in pharmaceutical care service's implementation. The health awareness programs were occasionally organized by almost all of the hospitals but unfortunately, pharmacists' participation was unsatisfactory. Doctors were more encouraged for their participation as compared to pharmacists. It could be of greater value for pharmacists to participate in such programs so that general public may know about pharmacists and pharmaceutical care services that supported the study carried out in Punjab Province of Pakistan [17], in which respondents suggested that expertise of pharmacists should be recognized by organization of health awareness programs.

A large number of the respondents admitted that they had found errors in the prescriptions like wrong doses, drug-drug interactions, and irrelevant drugs without any indication that can be reduced by the involvement of pharmacists due to their greater knowledge about drugs. They had further expressed that prescribing errors were one of the main reasons of adverse drug events that persistent with the study of Bates et al. in which they had demonstrated that nearly half of the preventable adverse drug events resulted from prescribing errors [18]. The main reason of prescribing errors was a communication gap between the pharmacists and the physician in relation to drug and patient at the time of prescription [19]. It can be minimized by incorporation of pharmacists into the patient-care team, as suggested in a Nigerian study [12], which revealed that pharmacists at various levels of hospitals can minimize the incidence of prescription errors and can optimize the medication therapy.

For successful implementation of pharmaceutical care services, there is a need to develop a bond between the pharmacist and the patient that was only possible through an effective communication. In qualitative part of study, the rate of provision of counseling services was low, which would strongly affect the patient adherence and compliance to the prescribed drug therapy. This finding was consistent with a study implemented in Kuwait [1], which also reported the low rate of counseling provision to patients leading to patients' noncompliance.

Another barrier reported was the insufficient number of pharmacists working in a pharmacy that was highlighted in a review [20], which also showed the shortage of pharmacists as the main hurdle in the implementation of Good Pharmacy Practice in Pakistan; as a result there was a work load on pharmacists. In the majority of public hospitals, pharmacists were limited to the hospital pharmacy. They were extremely involved in drug procurement, purchasing, and dispensing because of which they were unable to provide the pharmaceutical care services to patients. Pharmacists can focus on direct patient care if they are less involved in drug procurement and dispensing [21].

The quantitative part of the study revealed that a great proportion of the respondents were involved in patients counseling that was in line with an earlier quantitative study conducted in the Punjab Province of Pakistan [7], in which majority of the pharmacists were found to be involved in counseling.

## 4. Limitation of the Study

The present study involved hospital pharmacists working in only one province, Khyber Pakhtunkhwa, Pakistan. There is possibility that hospital pharmacists all over the country have a similar approach towards quality of pharmaceutical care services in the healthcare system, but results of this study may not be applicable to pharmaceutical care services in remaining regions of the country.

## 5. Conclusion and Recommendations

Majority of the findings of both qualitative and quantitative phases of this study are similar which revealed that hospital pharmacists in Pakistan are not actively participating in the provision of pharmaceutical care services. They are facing significant hurdles for their active participation in patient care; major obstacles are the unavailability of the sufficient number of pharmacists, lack of appropriate time for patient counseling, lack of participation in health awareness programs, and poor relationship between pharmacists and other health care providers. Moreover, there is a need to explore the pharmaceutical care concept among the other healthcare providers and general public by organizing pharmaceutical care awareness programs.

## Figures and Tables

**Table 1:** Demographic profile of respondents.

Characterization	Qualitative, *n* (%)	Quantitative, *n* (%)
Age range (years)		
20–30	3 (23.0)	90 (80.4)
31–40	8 (61.5)	19 (17.0)
41–50	2 (15.4)	3 (2.7)
Gender		
Male	9 (69.2)	93 (83)
Female	4 (30.7)	19 (17)
Years of practice		
<1	1 (7.7)	16 (14.3)
1–5	10 (76.9)	83 (74.1)
6–10	2 (15.4)	13 (11.6)
Type of hospital		
Public	7 (53.8)	31 (27.7)
Private	6 (46.2)	81 (72.3)

**Table 2:** Interaction with doctors on patients' medication.

Variable	*n* (%)
Doctors call for advice	
Never	21 (18.8)
Sometimes	75 (67.0)
Always	16 (14.3)
Contact with doctors regarding patient's prescription	
Never	13 (11.6)
Sometimes	89 (79.5)
Always	10 (8.9)
Reasons for interaction	
Drug availability	64 (57.1)
Side effects	16 (14.3)
Drug alternative	35 (31.3)
Drug dosage	33 (29.5)
Drug interactions	40 (35.7)
ADRs	19 (17)
Pharmacists' suggestions regarding drug related problems are considered by the physicians	
Never	21 (18.8)
Sometimes	69 (61.6)
Always	22 (19.6)

**Table 3:** Perception regarding patient counseling.

Items in questionnaire	Responses		*P* value^†^			
	Yes, *n* (%)	No, *n* (%)	Age	Gender	Type of hospital	Years of practice
Physicians are ready to accept your participation in patient counseling	70 (62.5)	42 (37.5)	0.004^*^	0.559	0.005^*^	0.786
Involved in educating patients	101 (90.2)	11 (9.8)	0.028^*^	0.464	0.000^*^	0.430
Spend enough time on each patient	55 (49.1)	57 (50.9)	0.436	0.179	0.027^*^	0.376
Inform about drug and food interactions	93 (83)	19 (17)	0.218	0.412	0.479	0.201
Instruct how to use their medications	103 (97.3)	3 (2.7)	0.686	0.427	0.824	0.584
Inform about side effects of drugs	82 (73.2)	30 (26.8)	0.342	0.026^*^	0.025^*^	0.716
Inform about storage conditions of drugs	99 (88.4)	13 (11.6)	0.799	0.028^*^	0.355	0.895

^†^Chi-square; ^*^significant relationship with the respective variables.

**Table 4:** Patients reporting of ADRs.

Items in questionnaire	Responses		*P* value^†^			
	Yes, *n* (%)	No, *n* (%)	Age	Gender	Type of hospital	Years of practice
Patients inform you about ADRs occurrence	35 (31.3)	77 (68.8)	0.280	0.973	0.442	0.706
Maintain the records of ADRs reporting	19 (17.0)	93 (83.0)	0.061	0.602	0.067	0.222
Patients are aware of ADRs reporting	11 (9.8)	101 (90.2)	0.373	0.114	0.975	0.430

^†^Chi-square.

**Table 5:** Participation in health awareness programs.

Items in questionnaire	Responses		*P* value^†^			
	Yes, *n* (%)	No, *n* (%)	Age	Gender	Type of hospital	Years of practice
Hospital organizes health awareness programs	83 (74.1)	29 (25.9)	0.138	0.535	0.152	0.962
Your participation in organization of such programs	63 (56.3)	49 (43.8)	0.215	0.392	0.129	0.857
Your satisfaction with these health awareness programs	47 (42.0)	65 (58.0)	0.082	0.989	0.390	0.455
Your participation is satisfactory	36 (32.1)	76 (67.9)	0.250	0.027^*^	0.987	0.739
Pharmacists are encouraged to participate in such health awareness programs	53 (47.3)	59 (52.7)	0.792	0.044^*^	0.777	0.611

^†^Chi-square; ^*^significant relationship with the respective variables.

**Table 6:** Perception regarding reducing prescribing errors.

Items in questionnaire	Responses					*P* value^†^			
	Always, *n* (%)	Often, *n* (%)	Sometimes, *n* (%)	Rarely, *n* (%)	Never, *n* (%)	Age	Gender	Type of hospital	Years of practice
Find the drug interaction problems	5 (4.5)	23 (20.5)	40 (35.7)	35 (31.3)	9 (8.0)	0.107	0.196	0.017	0.324
Find dose adjustment problems	7 (6.3)	30 (26.8)	46 (41.1)	19 (17.0)	10 (8.9)	0.126	0.249	0.194	0.065
Find the irrelevant drug prescriptions	4 (3.6)	26 (23.2)	41 (36.6)	25 (22.3)	16 (14.3)	0.012^*^	0.180	0.069	0.738

^†^Chi-square; ^*^significant relationship with the respective variables.
