# Corrigendum to “What Do the Hospital Pharmacists Think about the Quality of Pharmaceutical Care Services in a Pakistani Province? A Mixed Methodology Study”

**DOI:** 10.1155/2018/8705128

**Published:** 2018-02-27

**Authors:** Ghulam Murtaza, Rozina Kousar, Saira Azhar, Shujaat Ali Khan, Qaisar Mahmood

**Affiliations:** ^1^Department of Pharmacy, COMSATS Institute of Information Technology, Abbottabad 22060, Pakistan; ^2^Department of Environmental Sciences, COMSATS Institute of Information Technology, Abbottabad 22060, Pakistan

In the article titled “What Do the Hospital Pharmacists Think about the Quality of Pharmaceutical Care Services in a Pakistani Province? A Mixed Methodology Study” [1], the order of the first four references should be included as follows:

  [1] C. D. Hepler and L. M. Strand, “Opportunities and responsibilities in pharmaceutical care,”* The American Journal of Hospital Pharmacy*, vol. 47, no. 3, pp. 533–543, 1990.
  [2] N. E. Oguegbulu and I. F. Uche, “Patient-oriented re-professionalization of pharmacy practice; a sine qua non for the globally evolving collaborative practice of pharmaceutical medicine,”* International Journal of Pharmaceutical Sciences*, vol. 3, pp. 72–92, 2011.
  [3] J. A. Johnson and J. L. Bootman, “Drug-related morbidity and mortality and the economic impact of pharmaceutical care, ”*The American Journal of Health-System Pharmacy*, vol. 54, no. 5, pp. 554–558, 1997.
  [4] A. Awad, S. Al-Ebrahim, and E. Abahussain, “Pharmaceutical care services in hospitals of Kuwait,”* Journal of Pharmacy and Pharmaceutical Sciences*, vol. 9, no. 2, pp. 149–157, 2006.

Therefore, in Introduction, the text reading “The pharmacy profession is undergoing a paradigm shift from product-oriented to patient-oriented practice. This patient-oriented practice is termed as pharmaceutical care. Throughout the world, the principal task of pharmacy is considered to be the pharmaceutical care [1]. Pharmaceutical care means the responsible provision of drug therapy for achieving definite outcomes that improve the quality of patient's life [2]. Improved therapeutic outcomes include greater patient safety, better drug therapy and disease management, valuable health care expenditure, best adherence, and improved quality of life [3].

Pharmaceutical care practice involves a covenantal relationship between the pharmacist and the patient in which drug use is controlled by the pharmacist along with commitment and understanding of the patient's interest [2]. It is perceived as pharmacy profession's growth by accepting the social duty to diminish preventable drug-related morbidity and mortality [4].

Pharmaceutical care practice involves the periodic revolution in health care services provided by pharmacists. Several studies conducted in the developed countries have reported that pharmaceutical care practice has a considerable positive effect on health care cost and management [1] and pharmacists have worked a lot for implementation of pharmaceutical care practice” should be corrected as follows:

“The pharmacy profession is globally evolving from product-oriented practice to pharmaceutical care service [1]. It is a patient-oriented practice that is responsible for cost-effective provision of better drug therapy to achieve alleviated therapeutic effects leading to better disease management, greater patient safety, improved quality of patient life, best adherence, and valuable health care expenses [2,3].

Pharmaceutical care relates the patient's interest to the pharmacist's services through controlled use of medicines and useful counselling [2]. This periodically growing practice can be perceived as a social duty of pharmacist to prevent morbidity and mortality due to misuse of drugs [4]. It has been concluded from the pharmacy practice studies conducted in the developed countries that pharmacists have successfully implemented pharmaceutical care practice, exerting a substantial positive effect on health care expenses and management [1].”

Moreover, the article by Braun and Clarke [2] should be added as reference [22] in the original article.

Additionally, in Methods, the text reading “In quantitative phase of this study, questionnaire was developed for this cross-sectional survey on the basis of findings of qualitative phase. Moreover, literature was extensively reviewed [5, 9, 12, 13]” should be corrected to “After reviewing literature [5, 9, 12, 13], questionnaire was developed for this cross-sectional, quantitative study on the basis of findings of qualitative phase.”

Finally, in Methods, the text reading “In qualitative phase, an interview guide was developed after extensively reviewing the literature [1, 5, 9, 11] for execution of interviews. After getting appointment telephonically, the participants were interviewed directly by one of the researchers (RK), who is pharmacist, by using snow ball sampling technique in hospital pharmacies. Saturation point was reached after 13th interview.” should be updated as follows:

“In qualitative phase, an interview guide was developed after extensively reviewing the literature [1, 5, 9, 11] for execution of interviews. After getting appointment telephonically, the participants were interviewed directly by one of the researchers (RK), who is pharmacist, by using snow ball sampling technique in hospital pharmacies. The interviews were conducted in English, audio-taped, and transcribed verbatim. Saturation point was reached after 13th interview and no new theme was emerged in last two interviews. Transcription of interviews was double checked by respondents for its accuracy. Thematic content analysis of collected data yielded 5 major themes. Thematic analysis was performed by coding process in six phases to create meaningful patterns. These phases included familiarization with data, generating initial codes, searching for themes among codes, reviewing themes, defining and naming themes, and producing the final report [22].”
